# Innovative catheter modification for engaging an anomalous right coronary artery: a case report

**DOI:** 10.1093/ehjcr/ytag302

**Published:** 2026-05-13

**Authors:** Manoj Sharma, Mahmoud Elamin, Husam Katib, Ngoc Thai Kieu, Wassim Mosleh

**Affiliations:** Department of Internal Medicine, University at Buffalo, 955 Main St, Buffalo, NY 14203, USA; Department of Internal Medicine, University at Buffalo, 955 Main St, Buffalo, NY 14203, USA; Department of Internal Medicine, University at Buffalo, 955 Main St, Buffalo, NY 14203, USA; Department of Internal Medicine, University at Buffalo, 955 Main St, Buffalo, NY 14203, USA; Department of Interventional Cardiology, Mercy Hospital of Buffalo, Buffalo, NY 14220, USA

**Keywords:** Anomalous right coronary artery, Left sinus of Valsalva, NSTEMI (non-ST-elevation myocardial infarction), Coronary angiography, Percutaneous coronary intervention, Femoral access, Radial access, Guide catheter modification, Case reports

## Abstract

**Background:**

Anomalous right coronary artery (RCA) arising from the left sinus of Valsalva presents a challenge during angiography, often leading to failed engagement, prolonged procedure time, and delayed reperfusion. This case highlights an innovative technique in which a standard guide catheter was manually modified to mimic a Leya catheter when the dedicated device was unavailable, allowing timely revascularization.

**Case summary:**

A 47-year-old man presented with chest pain and elevated troponin, consistent with non-ST-elevation myocardial infarction. Angiography showed an anomalous RCA taking off anterior and superior to the left main coronary artery. Several standard diagnostic and guiding catheters failed to engage the vessel; the procedure was then switched from radial to femoral access, and an Amplatz Left 1 (AL1) guide catheter was manually modified by applying a 45–60° right-angled twist at the tip to reproduce the curvature of a Leya catheter. This modification allowed stable engagement of the RCA and successful percutaneous coronary intervention.

**Conclusion:**

In patients with anomalous RCA where standard catheters fail, manual modification of a guide catheter to achieve optimal engagement can be a useful alternative when a dedicated design is unavailable. Femoral access is preferred in difficult cases, especially with anomalous RCA, as it provides greater stability and facilitates successful engagement.

Learning points

- Engagement of anomalous RCA may be optimized by adapting an early procedural strategy such as access site and catheter geometry in order to avoid unnecessary delays in reperfusion.
- If guides are unavailable, manual modification of a standard catheter can provide the necessary coaxial alignment and support to safely complete PCI in complex congenital anatomy.

## Introduction

Congenital coronary artery anomalies (CAAs) occur in ∼0.2%–1.2% of patients undergoing percutaneous coronary intervention (PCI).^1^ These anomalies can make angiographic intervention more difficult.^2,3^ Often requiring increasing contrast and longer fluoroscopy times delaying treatment. If unrecognized, it may be mistaken for an occluded artery, leading to potential diagnostic error.^2,4,5^ Understanding these variants is crucial for procedural planning to help ensure timely engagement, optimal imaging, and successful PCI when needed.^1,6^

**Figure 1:**
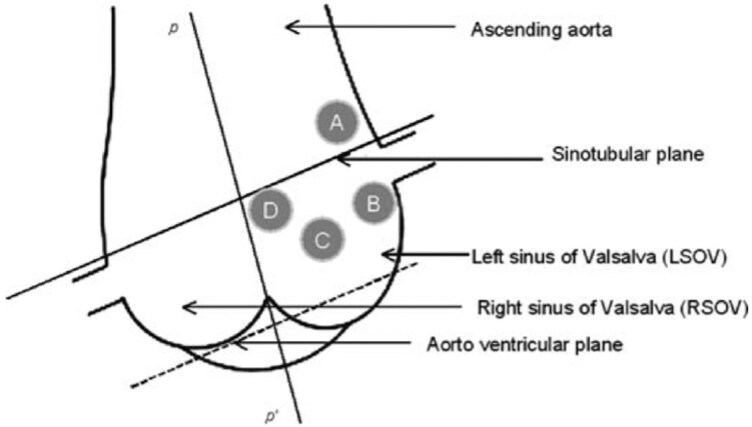
Origin of anomalous RCA from LSOV. Representative diagram of the aortic root and sinuses in left anterior oblique (LAO) projection. P–P′ indicates a hypothetical plane running through the midline. Sites A through D represent common sites for the origin of the anomalous artery. Reproduced with permission from Sarkar K, Sharma SK, Kini AS. Journal of Interventional Cardiology. 2009;22(3):234–239. © Wiley.^1^

Anomalous origin of the right coronary artery (AORCA) from the left sinus of Valsalva (LSOV) accounts for roughly 6%–27% of all reported coronary variants and is associated with sudden cardiac arrest, myocardial ischaemia, ventricular arrhythmias, and syncope, making individualized interventional planning essential.^1,6^ Uthayakumaran *et al.* described four typical sites of origin around the LSOV and left coronary artery (LCA), proposing a practical classification system (*Figure 1*).^6–8^ These include a take-off just above the LCA ostium, designated A in *Figure 1*, just below it (B), and along the midline of the ascending aorta (C and D).^1,6^ Its unusual take-off complicates angiography and demands careful anatomical review before intervention.

## Summary figure

**Figure:**
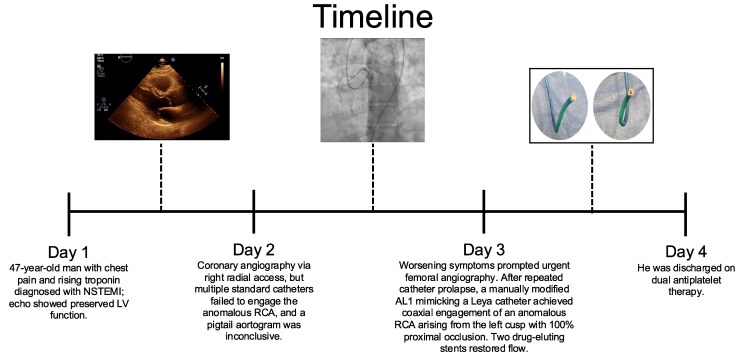


## Case presentation

A 47-year-old man with hyperlipidaemia and a 30-pack-year smoking history presented with sudden onset chest pain radiating to his left shoulder. He described numbness in the left arm, shortness of breath, diaphoresis, and exertional chest discomfort. On arrival, his blood pressure was 193/139 mmHg. Cardiovascular examination revealed a regular rate and rhythm without murmurs, rubs, or gallops. There was no jugular venous distension and no peripheral oedema.

Laboratory tests showed a normal complete blood count and metabolic profile. High-sensitivity troponin was elevated, peaking at 8449 pg/mL (normal <15 pg/mL). The electrocardiography demonstrated non-specific ST-segment changes with an incomplete right bundle-branch block, and the chest radiography was unremarkable. He was diagnosed with a non-ST-elevation myocardial infarction (NSTEMI) and was started on an acute coronary syndrome protocol. The transthoracic echocardiography revealed preserved left ventricular systolic function (60%–65%) without regional wall-motion abnormalities.

Initial coronary angiography was performed via the right radial artery with multiple standard diagnostic catheters including JR4, 3DRC, AL1, MPA, Tiger, Jacky, and an AR1 guide which failed to engage the right coronary artery (RCA). A pigtail aortogram was inconclusive, and because of the large contrast load, the procedure was paused in favour of a planned coronary CT angiogram (CCTA).

**Figure 2:**
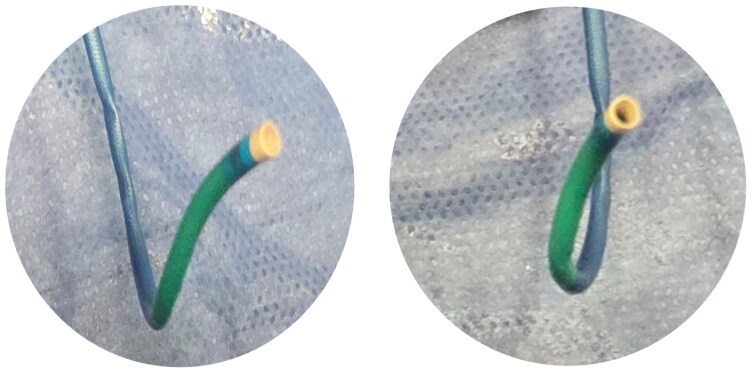
A manually modified AL1 with the tip reshaped with a 45–60° right-angled twist to mimic the Leya configuration.

Worsening symptoms prompted an urgent angiogram performed via the right femoral approach. Repeated prolapse into the left main coronary artery (LCMA) limited device delivery. The RCA was successfully engaged using a 5 Fr Jacky diagnostic catheter, which is normally a transradial-specific catheter. This confirmed the anomalous origin of the RCA from the left coronary cusp, which appeared anterior and slightly cephalad to the left main artery (Type A). Because dedicated anomalous RCA guides were unavailable, the AL1 catheter was manually modified with a 45–60° right-angled twist under sterile technique (*Figure 2*). This provided coaxial engagement, and a 6 Fr GuideLiner was advanced into the proximal RCA to maintain stability as the catheter warmed and attempted to regain its original shape.

After several attempts, the RCA was engaged confirming an anomalous origin of the RCA from the left coronary cusp. It was located anterior and slightly cephalad to the left main artery (Type A). A 100% occlusion was noted in the proximal-to-mid RCA segment, and two drug-eluting stents were placed that restored flow through the vessel. He was discharged on dual antiplatelet therapy with aspirin and clopidogrel (see Supplementary material online, *Videos S1*–*S4*).

## Discussion

We demonstrated how anomalous coronary arteries can present a distinctive challenge, where timely and effective revascularization is critical. Usually, the coronary ostia are circular, located below the sinotubular ridge, within the sinus of Valsalva, centrally located between the commissural attachments of the aortic cusps.^1,6,7,9,10^ With the anomalous RCA, the ostium usually is slit-like, commonly lies anterior and cephalad to LCA, and the artery takes an acute rightward course that precludes the coaxial engagement of most of the currently available guiding catheters. The risk of coronary dissection increases when using an inappropriate guide catheter, and non-coaxial engagement of the anomalous vessel may lead to decreased support, resistance to delivery of hardware, and inadequate angiographic visualization. Moreover, an inadequate backup force may lead to PCI failure.^9,10^

PCI of anomalous RCA often requires multiple catheter exchanges, prolonging procedure time and increasing ischaemic risk.^1,6^ These patients may also be predisposed to endothelial dysfunction and accelerated atherosclerosis.^5,7,8,11,12^ When standard engagement fails, interventionalists should consider alternative strategies such as specialized catheters, deeper guide support, and adjunctive intracoronary imaging.

### Anomalous right coronary artery origins: anatomical variants and catheterization strategies

The RCA may arise anomalously from various locations within the sinuses of Valsalva, each presenting distinct technical challenges for catheter engagement. These variants include as follows: (A) adjacent to the LMCA ostium, (B) inferior to the LMCA ostium, (C) near the commissure between right and left coronary cusps, (D) above the sinotubular junction, (E) from the right cusp directed superiorly and leftward, and (F) the usual origin.^1,2,4,6,13^

Location A is the most frequent and technically challenging. Optimal engagement can be achieved using either a 7 F Leya left coronary Amplatz 45 or 90° catheter (Cordis) or a 6 F R-ACAOS Launcher guide catheter (Medtronic).^1,6,10^ This variant often includes a short intramural segment that can be compressed during systole. This feature warrants particular care during catheter manipulation to prevent iatrogenic dissection. Locations B and C are typically easier to access and can be engaged with Amplatz left or right catheters (Types 1–3), selected according to aortic root dimensions. In contrast, Location D resembles the course of a saphenous vein graft and can be approached using Judkins right 4, hockey stick, multipurpose, or Amplatz catheters.^12^ When selective engagement is not possible, the guide may be placed near the ostium, allowing a wire to be advanced first, followed by a guide-extension catheter to support intervention. In our case, the initial radial approach failed to engage the RCA because of its anterior and rightward take-off; switching to femoral access provided greater control and ultimately enabled successful engagement.^2,13^

Femoral access is traditionally preferred in the setting of anomalous coronary anatomy providing advantages such as guidewire manoeuvrability, decreased torque resistance, and enhanced backup support and facilitates complex adjunctive manoeuvres such as balloon anchoring. Left radial artery access may be advantageous in selected cases of anomalous right coronary artery engagement by reducing vascular complications and improving patient comfort. Access site selection should therefore be individualized based on coronary anatomy, procedural complexity, and operator experience.

A specialized catheter is needed when standard guiding catheters prove inadequate but are often unavailable in most laboratories.^1,6^ We demonstrated that it is possible to manually modify guides, such as the AL1 guide catheter to mimic the Leya catheter, facilitating successful PCI. Several alternative techniques have been described for engaging anomalous RCA origins. Sarkar *et al*. reported success with modified Judkins left (JL) and Amplatz left (AL) catheters. Lorin *et al*.^14^ described effective PCI using a JL 5.0 guiding catheter via the radial route after unsuccessful femoral engagement. Another described technique is the ‘swan-neck manoeuvre’, in which a Judkins catheter is reshaped to allow selective cannulation.^15,16^

## Conclusion

Our case builds on the work done by Sarkar *et al.*, showing how a modified guide catheter can achieve successful engagement when dedicated anomalous RCA guides are not available. This evolution underscores the importance of procedural flexibility and innovation.

## Supplementary Material

ytag302_Supplementary_Data

## Data Availability

The data underlying this article are available in the article and its online Supplementary material. Further inquiries can be directed to the corresponding author.

## Lead author biography

Manoj Sharma, MD, is an Internal Medicine resident at the University at Buffalo. He is dedicated to growing as a thoughtful, reliable clinician and values the relationships built through patient care. Over the course of his training, he has developed a strong interest in cardiology, especially in the way small decisions can profoundly change patient outcomes. He hopes to continue building his clinical skills and pursue a career in cardiology in the future.

## Notes

**Consent:** The authors confirm that written consent for submission and publication of this case report including images and associated text has been obtained from the patient, in line with COPE guidance. Written informed consent was obtained from the patient for publication of this case report and accompanying images.
